# Author Correction: Inferring disease subtypes from clusters in explanation space

**DOI:** 10.1038/s41598-024-56360-3

**Published:** 2024-03-14

**Authors:** Marc-Andre Schulz, Matt Chapman-Rounds, Manisha Verma, Danilo Bzdok, Konstantinos Georgatzis

**Affiliations:** 1 Department of Psychiatry, Psychotherapy and Psychosomatics, RWTH Aachen University, Aachen, Germany; 2 Department of Psychiatry and Neurosciences, Charité – Universitätsmedizin Berlin, corporate member of Freie Universität Berlin and Humboldt-Universität zu Berlin, Berlin, Germany; 3 School of Informatics, University of Edinburgh, Edinburgh, UK; 4 Present Address: Verizon Media, Boston, MA USA; 5 Department of Biomedical Engineering, McConnell Brain Imaging Centre (BIC), Montreal Neurological Institute (MNI), Faculty of Medicine, McGill University, Montreal, Canada; 6 Mila - Quebec Artificial Intelligence Institute, Montreal, Canada; 7 QuantumBlack, London, UK

Correction to: *Scientific Reports* 10.1038/s41598-020-68858-7, published online 30 July 2020

The original version of this Article omitted an affiliation for author Marc-André Schulz. The correct affiliations are listed below.

Department of Psychiatry, Psychotherapy and Psychosomatics, RWTH Aachen University, Aachen, Germany

Department of Psychiatry and Neurosciences, Charité – Universitätsmedizin Berlin, corporate member of Freie Universität Berlin and Humboldt-Universität zu Berlin, Berlin, Germany

QuantumBlack, London, UK

The original Article has been corrected.

